# Therapeutic effects of an oral gonadotropin‐releasing hormone receptor antagonist, relugolix, on preventing premature ovulation in mild ovarian stimulation for IVF

**DOI:** 10.1002/rmb2.12422

**Published:** 2021-10-19

**Authors:** Kazuki Nakao, Keiji Kuroda, Takashi Horikawa, Azusa Moriyama, Hiroyasu Juen, Akiko Itakura, Hideaki Watanabe, Satoru Takamizawa, Yuko Ojiro, Koji Nakagawa, Rikikazu Sugiyama

**Affiliations:** ^1^ Center for Reproductive Medicine and Implantation Research Sugiyama Clinic Shinjuku Tokyo Japan; ^2^ Department of Obstetrics and Gynecology Faculty of Medicine Juntendo University Tokyo Japan

**Keywords:** Gonadotropin‐releasing hormone receptor antagonist, in vitro fertilization, mild ovarian stimulation, premature ovulation, relugolix

## Abstract

**Purpose:**

Can relugolix, a novel oral gonadotropin‐releasing hormone receptor (GnRH) antagonist, function as an alternative ovulation inhibitor to GnRH antagonist injections?

**Methods:**

This single‐center, cross‐sectional retrospective study compared premature ovulation rates and clinical outcomes in IVF treatment after mild ovarian stimulation with 40 mg of relugolix (relugolix group) or 0.25‐mg injections of ganirelix acetate or cetrorelix acetate (injection group) between March 2019 and January 2020. Of 247 infertile women (256 IVF cycles) aged ≤42 years, 223 women (230 cycles) were evaluated. In the relugolix and injection groups, we compared 104 and 85 cycles after GnRH antagonist use before the LH surge (LH levels <10 mIU/ml) and 22 and 19 cycles during the LH surge (LH levels ≥10 mIU/ml), respectively.

**Results:**

Before the LH surge, the ovulation rates in the two groups were very low (*p* = 0.838), however; during the LH surge, the cycles using relugolix had a high ovulation rate of 40.9% compared with no ovulation in the injection group (*p* = 0.002). There were no significant differences in embryo culture findings and pregnancy outcomes between the two groups.

**Conclusions:**

Although relugolix had a high ovulation suppressive effect, when the LH surge occurred, its effect was insufficient.

## 1 INTRODUCTION

Ovarian stimulation is necessary in assisted reproductive technology for recruiting multiple fertilizable oocytes from a sufficient size and number of follicles. The timing of final oocyte maturation using human chorionic gonadotropin (hCG) injection as a trigger and timed follicle aspiration immediately before ovulation are also important for collecting mature eggs in the follicles during oocyte retrieval.^1^ Mild ovarian stimulation for IVF treatment has become common throughout the world as a safer, more affordable, and more patient‐friendly protocol compared with conventional ovarian stimulation, such as gonadotropin‐releasing hormone (GnRH) agonist and antagonist protocols.^2^, ^3^ It essentially uses lower doses or shorter durations of gonadotropin administration without GnRH analogs; therefore, the control of premature ovulation prior to oocyte retrieval is required.^2^ During the mild or modified natural stimulation protocol, procedures for suppressing ovulation include use of nonsteroidal anti‐inflammatory drugs (NSAIDs).^4^ However, its suppressive effect is not strong and does not last long.^4^

A GnRH receptor antagonist has strong effectiveness for suppressing the release of endogenous gonadotropins from the pituitary, and its effects are initiated in a short period of time^5^, ^6^, ^7^; therefore, it is frequently used during controlled ovarian stimulation as a GnRH antagonist protocol. Although a GnRH antagonist agent can be used in mild ovarian stimulation,^8^ the conventional GnRH antagonist is administered via injection and is generally expensive, placing physical and financial burdens on the patients.

In recent years, a novel oral GnRH antagonist medication, relugolix, was launched as a therapeutic agent for uterine fibroids and endometriosis.^9^, ^10^ Relugolix intake is easier and less invasive than an injection agent^10^; therefore, it is expected to function as an alternative ovulation inhibitor during ovarian stimulation. This study aimed to evaluate an ovulation suppressive effect of relugolix after mild ovarian stimulation and the clinical outcomes in IVF treatment with the use of relugolix compared with conventional GnRH antagonist injection agents.

## 2 MATERIALS AND METHODS

### 2.1 Study design

**FIGURE 1:**
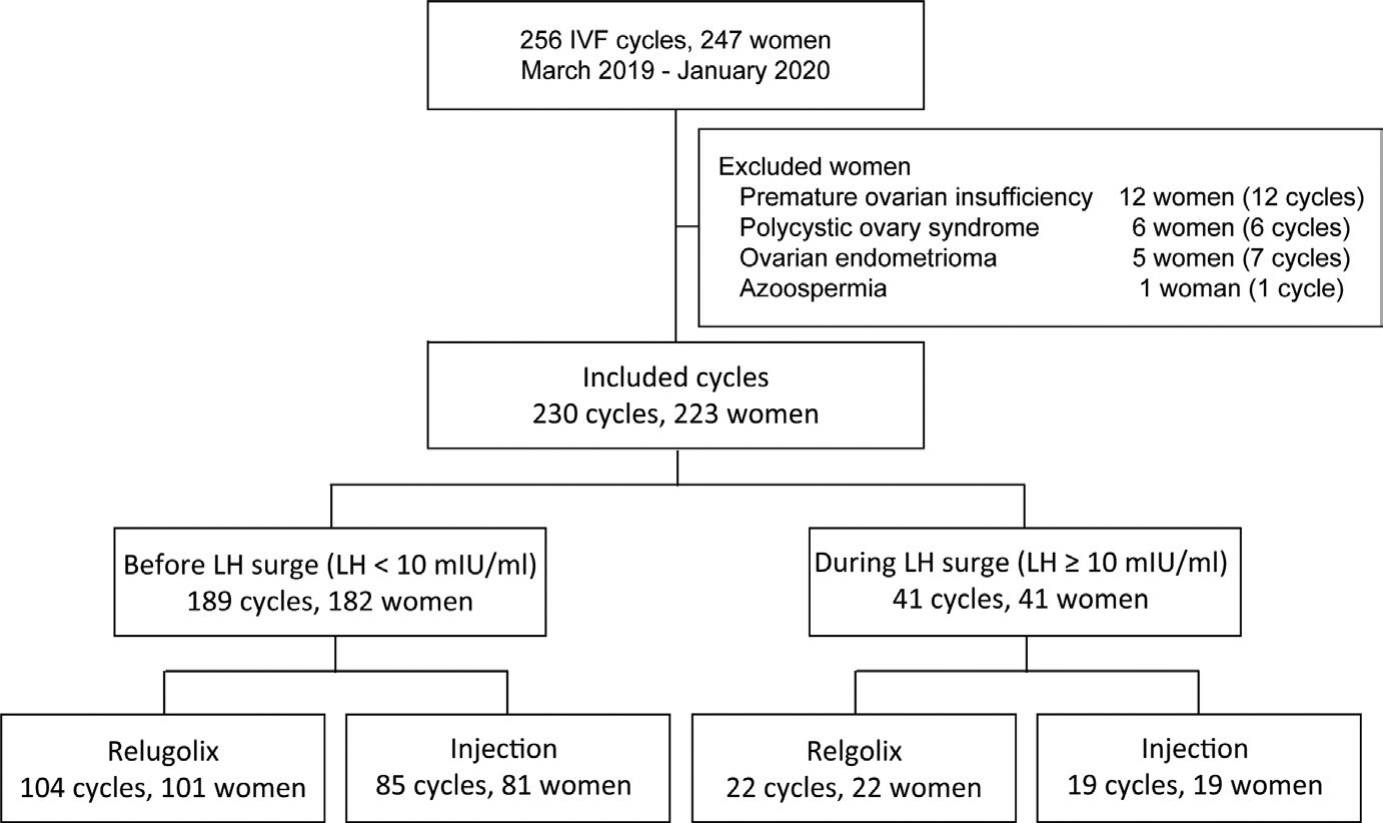
Flowchart of IVF cycles for analyzing the findings of oocyte retrieval. We compared IVF treatment cycles after mild ovarian stimulation using 40 mg of oral relugolix (relugolix group) and 0.25‐mg injections of ganirelix acetate or cetrorelix acetate (injection group) between March 2019 and January 2020. Of 256 IVF cycles in 247 infertile women aged ≤42 years, we evaluated 223 women (230 cycles) after excluding 18 women (18 cycles) with elevated basal LH levels including 12 with premature ovarian insufficiency and six with polycystic ovary syndrome; we also excluded six women (eight cycles) with factors influencing the outcomes of IVF treatments including five with ovarian endometriomas and one with a husband with azoospermia after testicular sperm extraction. In the relugolix and injection groups, we compared 104 and 85 cycles after GnRH antagonist use before the start of the LH surge (LH levels <10 mIU/ml) and 22 and 19 cycles during the LH surge (LH levels ≥10 mIU/ml), respectively

This study was a single‐center, cross‐sectional retrospective study that evaluated the clinical application of relugolix as an ovulation inhibitor. We analyzed premature ovulation rate in mild ovarian stimulation using relugolix as a primary outcome and clinical outcomes in IVF treatment after relugolix use as a secondary outcome. This study was conducted after all patients provided informed consent. We compared IVF treatment cycles after mild ovarian stimulation using 40 mg of oral relugolix (Relmina^®^ Tablets 40 mg, Asuka Pharmaceuticals) (relugolix group) or using 0.25‐mg injections of ganirelix acetate (Ganirest^®^ subcutaneous 0.25‐mg syringes, MSD Co., Ltd., Tokyo, Japan) or cetrorelix acetate (Cetrotide^®^ injection of 0.25 mg, Merck Biopharma Co., Ltd.) (injection group) between March 2019 and January 2020 in Sugiyama Clinic Shinjuku. Of the 256 IVF cycles in 247 infertile women aged 42 years or younger, we excluded 18 women (18 cycles) with elevated basal luteinizing hormone (LH) levels including 12 with premature ovarian insufficiency and six with polycystic ovary syndrome (PCOS); we also excluded six women (eight cycles) with factors that can influence the outcomes of IVF treatments including five with ovarian endometriomas and one with a husband with azoospermia (Figure 1). Therefore, 223 women (230 cycles) were evaluated. The incidence of premature LH surge was defined as 10 mIU/ml or more, and the relugolix and injection groups were compared separately by GnRH antagonist administration before and during LH surge. In the relugolix and injection groups, we compared 104 cycles (101 women) and 85 cycles (81 women) when the follicles were sufficiently developed after ovarian stimulation with GnRH antagonist use before LH surge initiation (LH levels <10 mIU/ml) and 22 cycles (22 women) and 19 cycles (19 women) during the LH surge (LH levels ≥10 mIU/ml), respectively.

**FIGURE 2:**
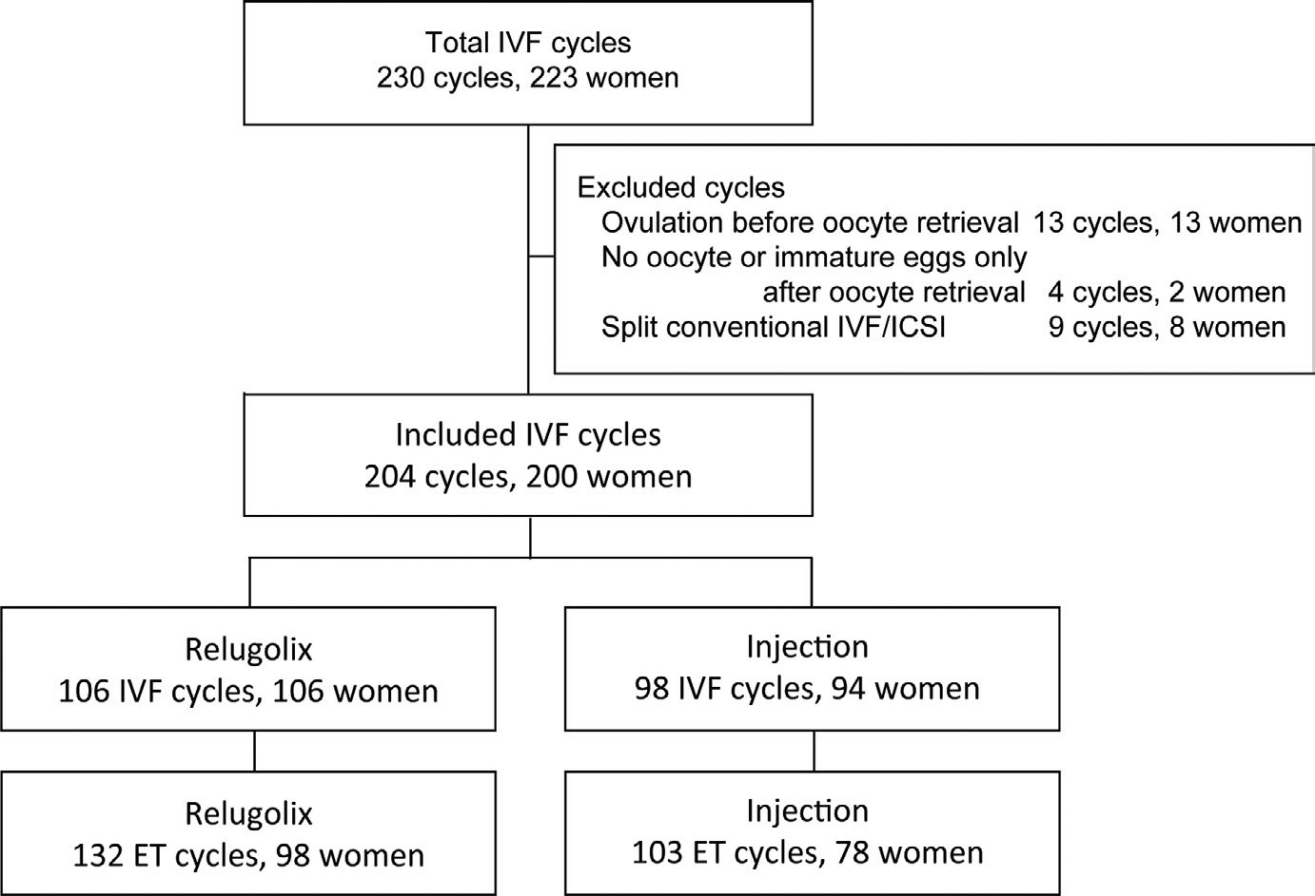
Flowchart of IVF cycles for the analysis of embryo culture findings and pregnancy outcomes. Of 230 IVF cycles, we evaluated 204 cycles after excluding 13 with premature ovulation, four without mature eggs after oocyte retrieval and nine with split conventional IVF and intracytoplasmic sperm injection (ICSI) as a fertilization method. We compared the findings of fertilization and embryo culture in 106 IVF cycles (106 women) and 98 IVF cycles (94 women) in patients who received oral relugolix or an injection of ganirelix or cetrorelix, respectively. Furthermore, pregnancy outcomes were compared between 132 ET cycles (98 women) and 103 ET cycles (78 women) in patients who received oral medicine and GnRH antagonist injections, respectively

To confirm the findings on fertilization, embryo culture, and pregnancy outcomes after using relugolix, we evaluated 204 of the 230 IVF cycles after excluding 13 cycles with premature ovulation before oocyte retrieval, four cycles without mature eggs after oocyte retrieval and nine cycles with split conventional IVF and intracytoplasmic sperm injection (ICSI) as a fertilization method (Figure 2). We compared the findings of fertilization and embryo culture in 106 IVF cycles (106 women) and 98 IVF cycles (94 women) after administering oral relugolix and ganirelix or cetrorelix injections, respectively. Furthermore, pregnancy outcomes were compared between 132 embryo transfers (ET) cycles (98 women) and 103 ET cycles (78 women) in patients who received oral medicine and GnRH antagonist injections, respectively. This study was approved by the local ethics committee of Sugiyama Clinic, Tokyo, Japan (No. 20–005).

### 2.2 Mild ovarian stimulation using GnRH antagonists and subsequent IVF‐ET

The ovarian stimulation, oocyte retrieval, and IVF‐ET treatment methods have been previously described.^11^, ^12^ Briefly, the women were administered 50–100 mg of clomiphene citrate (Clomid^®^; Fuji Pharma, Tokyo, Japan) once or twice daily for 5–10 days or 2.5 mg of letrozole (Kobayashi Kako Co., Ltd., Fukui, Japan) daily for 5 days beginning on day 3 of the menstrual cycle in combination with the injection of 150–225 IU per day of recombinant FSH (Gonal‐f^®^; Merck Biopharma Co., Ltd.) or hMG (HMG Ferring; Ferring Pharmaceuticals) on days 3, 4, 6, and 8.

On menstrual day 8–10, when the dominant follicles had reached 16 mm in size or larger before the start of the LH surge, the once daily administrations of 40 mg of oral GnRH antagonist tablets (relugolix) or 0.25 mg of ganirelix acetate or cetrorelix acetate and 225 IU per day of recombinant FSH or hMG were initiated until the dominant follicles reached >17 mm. Then, we administered either 250 µg of a recombinant hCG injection (Ovidrel^®^; Merck Biopharma Co., Ltd.) for oocyte maturation.

Or, on menstrual day 9–11, when the dominant follicles had reached 17 mm or larger during the LH surge, 40 mg of an oral relugolix tablet or 0.25 mg of a GnRH antagonist injection was administered on the day of trigger administration for oocyte maturation with the use of hCG. Having food diminishes the bioavailability of relugolix^9^; therefore, we recommended taking relugolix before meals. NSAIDs were not used for the purpose of suppressing ovulation in all cycles. Oocyte retrieval was performed transvaginally at 35 h after trigger administration. Ovulation was defined as the rupture of one or more follicles prior to oocyte retrieval.

Either conventional IVF or ICSI was chosen based on the sperm findings and previous fertilization rates. Oocyte maturation was determined by the appearance of the first polar body. Fertilization was confirmed by the detection of two pronuclei and the second polar body. All embryos were evaluated by the Veeck classification at the cleavage stage and the Gardner classification at the blastocyst stage. Morphologically good blastocysts were defined as 5‐ or 6‐day blastocysts of stage 4 or more and grade A or B in both the inner cell mass and the trophectoderm of the Gardner classification.

In our protocol, fresh ET using a cleavage stage embryo and freezing excess blastocysts are mainly performed after ovarian stimulation with letrozole and FSH or hMG injections in the first oocyte retrieval cycle. In the second or later cycles, we performed all blastocysts were cryopreserved using the vitrification method after clomiphene and FSH or hMG and subsequently single blastocyst transfer, because the live birth rate after frozen ET is significantly higher than that in fresh ET.^13^ Fresh ET was performed using a soft catheter (Kitazato ET catheter; Kitazato Corporation, Shizuoka, Japan) guided by a transvaginal ultrasound 2, 3, or 5 days after conventional IVF or ICSI. As luteal support, 30 mg of oral dydrogesterone (Dufaston^®^ Tablets 5 mg; Mylan EPD LLC., Tokyo, Japan) was administered three times daily for 11 days from the day of ET, and 125 mg of hydroxyprogesterone caproate (Progeston^®^ depot intramuscular injection; Fuji Pharma) was injected on the day of ET and 5 days after ET. In the vitrified‐warmed ET cycles, the endometrium was prepared via either spontaneous ovulatory or hormone replacement cycles. In the spontaneous ovulatory cycle, the day of ET was defined as 2, 3, or 5 days after ovulation depending on the development stage of the frozen embryos. The same luteal support using dydrogesterone and hydroxyprogesterone caproate as that for the fresh ET cycle was used. In the hormone replacement cycle, 1.25 mg of conjugated estrogen tablets administered daily (Premarin^®^ 0.625 mg; Wyeth., Tokyo, Japan) and 2.88 mg of a transdermal estradiol patch (Estrana^®^ Tape 0.72 mg; Hisamitsu Pharmaceutical, Tokyo, Japan) administered every other day were started on day 3 of the menstrual cycle. When the endometrium was 7 mm or thicker on day 13, 30 mg of dydrogesterone tablets and 90 mg of OneCrinone^®^ (Vaginal progesterone gel; Merck Biopharma Co., Ltd.) were administered daily beginning on day 14 as luteal support. Clinical pregnancy was diagnosed with the detection of a gestational sac using a transvaginal ultrasound at 5 weeks of gestation (16–19 days after ET). A miscarriage was defined as pregnancy loss during clinical pregnancy between 5 and 12 weeks of gestation.

### 2.3 Statistical analysis

The statistical analyses were performed using GraphPad Prism 5 (GraphPad Software Inc.). Differences between two groups were analyzed using the Mann–Whitney *U* test or Fisher's exact test, as appropriate. The level of significance was defined as a *p* value of <0.05.

## 3 RESULTS

### 3.1 Use of GnRH antagonist agents before the start of the LH surge

To identify the therapeutic effect of relugolix for suppressing premature ovulation, we conducted comparisons between 104 cycles using relugolix (relugolix group) and 85 cycles with conventional GnRH antagonist injection (injection group) before the LH surge (LH levels <10 mIU/ml). The characteristics of the cycles are shown in Table 1. Although more women had ovulation disorders in the relugolix group than in the injection group, there were no significant differences in patients' age, pregnancy history, other causes of infertility, previous history of ET cycles, AMH levels, ovarian stimulation with and without clomiphene citrate, oocyte maturation procedure, or hormone data at the day of trigger administration. In our clinic, the costs of 40 mg of relugolix and a 0.25‐mg injection of a GnRH antagonist were JPY1,000 and 8,000 (USD9.1 and 72.9), respectively. Although there was no significant difference in the number of doses of GnRH antagonist tablet or injection, the cost for ovulation suppression was significantly lower in the relugolix group than in the injection group (USD13.7 ± 7.2 and 120.1 ± 66.8, respectively, *p *< 0.001). The ovulation rates in the relugolix and injection groups were very low (1.9% and 2.4%, respectively) and did not differ significantly (*p* = 0.838). In the oocyte retrieval cycles without premature ovulation, there were no significant differences in the number of retrieved oocytes and their oocyte maturation rates. No women complained of side effects after relugolix use.

**TABLE 1:** Characteristics and ovulation rates in oral relugolix and GnRH antagonist injection before LH surge

	Relugolix 104 cycles (101 women)	Injection 85 cycles (81 women)	*p*‐value
Age, years, mean ±SD (range)	35.9 ± 4.0	36.8 ± 3.5	0.114^a^
Pregnancy history, median (range)			
Gravidity	0 (0–5)	0 (0–3)	0.497^a^
Parity	0 (0–2)	0(0–2)	0.276^a^
Causes of infertility, n (%)			
Ovulation disorder	22 (21.2)	5 (5.9)	**0.003** ^b^
Tube factor	14 (13.5)	16 (18.8)	0.316^b^
Uterus factor	14 (13.5)	8 (9.4)	0.388^b^
Male factor	34 (32.7)	34 (40.0)	0.298^b^
Unexplained infertility	35 (33.7)	26 (30.6)	0.654^b^
Previous history of embryo transfer cycles, median (range)	0 (0–10)	1 (0–12)	0.521^a^
AMH, ng/ml, mean ± SD	4.4 ± 3.9	3.5 ± 2.5	0.051^a^
Ovarian stimulation, cycle, n (%)			
Clomiphene citrate + FSH/hMG	68 (65.4)	51 (60.0)	0.454
Letrozole + FSH/hMG	36 (34.6)	34 (40.0)	
Hormone data at the day of trigger administration, mean ± SD			
LH, mIU/ml	3.3 ± 2.6 (0.2–9.9)	3.7 ± 2.3 (0.5–9.2)	0.322^a^
Estradiol, pg/ml	1340 ± 889	1265 ± 913	0.569^a^
Progesterone, ng/ml	0.6 ± 0.4	1.0 ± 1.8	0.061^a^
Total dose of GnRH antagonist administration, mg, mean ± SD	60.2 ± 31.6	0.4 ± 0.2	–
The number of doses of GnRH antagonist, times, mean ± SD	1.5 ± 0.8	1.6 ± 0.9	0.811^a^
Cost for ovulation suppression^c^, mean ± SD	USD13.7 ± 7.2 (JPY1,504 ± 789)	USD120.1 ± 66.8 (JPY13,182 ± 7,330)	**<0.001** ^a^
Total ovulation rate, cycle, n (%)	2 (1.9)	2 (2.4)	0.838^b^
Clomiphene citrate +FSH/hMG	2 (2.9)	2 (3.9)	1.000^b^
Letrozole +FSH/hMG	0 (0)	0 (0)	1.000^b^
Findings of retrieved oocytes			
No. of retrieved oocytes, n, mean ± SD	7.6 ± 4.5	8.8 ± 6.2	0.222^a^
No. of retrieved mature oocytes, n, mean ± SD	5.9 ± 3.6	6.6 ± 4.6	0.231^a^
Oocyte maturation rate /oocyte, %, mean ± SD	76.7 ± 22.5	76.6 ± 21.9	0.969^a^

Abbreviations

AMH, anti‐Müllerian hormone; SD, standard deviation.

Bold indicates statistical significance *p* < .05.

^a^ Student's *t*‐test.

^b^ Fisher's exact test.

^c^ Converted to USD from JPY using average USD exchange rate in September 2021 published by Bank for International Settlements (JPY109.8/USD).

### 3.2 Use of GnRH antagonist agents during the start of the LH surge

When the LH surge was confirmed (LH levels ≥10 mIU/ml), premature ovulation was suppressed using relugolix (relugolix group) in 22 cycles and using GnRH antagonist injection (injection group) in 19 cycles at the day of trigger administration. As shown in Table 2, there were no significant differences in characteristics between the two groups. The cost for ovulation suppression using relugolix was significantly cheaper compared with that for the GnRH antagonist injection (USD9.1 ± 0 and 72.9 ± 0, respectively, *p* < 0.001). Surprisingly, the cycles using relugolix had a high ovulation rate of 40.9% compared with no cancellation of oocyte retrieval cycles in the injection group (*p* = 0.002). Of the 11 cycles after ovarian stimulation with clomiphene citrate, premature ovulation occurred with high frequency (six cycles, 54.5%). However, after oocyte retrieval, there were no significant differences in the findings of retrieved oocytes. No women experienced side effects with relugolix use.

**TABLE 2:** Characteristics and ovulation rates in oral relugolix and GnRH antagonist injection during LH surge

	Relugolix 22 cycles (22 women)	Injection 19 cycles (19 women)	*p*‐value
Age, years, mean ± SD (range)	36.0 ± 3.3	36.0 ± 4.9	0.719^a^
Pregnancy history, median (range)			
Gravidity	0 (0–1)	0 (0–3)	0.953^a^
Parity	0 (0–1)	0(0–1)	0.882^a^
Causes of infertility, n (%)			
Ovulation disorder	2 (9.1)	0 (0)	0.178^b^
Tube factor	2 (9.1)	2 (10.5)	0.877^b^
Uterus factor	3 (13.6)	5 (26.3)	0.144^b^
Male factor	7 (31.8)	7 (36.8)	0.735^b^
Unexplained infertility	8 (36.4)	7 (36.8)	0.975^b^
Previous history of embryo transfer cycles, median (range)	0 (0–5)	0 (0–4)	0.632^a^
AMH, ng/ml, mean ± SD	2.6 ± 2.8	2.5 ± 2.0	0.404^a^
Ovarian stimulation			
Clomiphene citrate + FSH/hMG	11 (50.0)	10 (52.6)	1.000
Letrozole + FSH/hMG	11 (50.0)	9 (47.4)	
Hormone data at the day of trigger administration, mean ± SD			
LH, mIU/ml	16.3 ± 5.3 (10.3–29.5)	16.2 ± 4.4 (10.0–24.8)	0.940^a^
Estradiol, pg/ml	619 ± 485	627 ± 715	0.570^a^
Progesterone, ng/ml	0.9 ± 1.8	1.0 ± 1.3	0.854^a^
Total dose of GnRH antagonist administration, mg, mean ± SD	40.0 ± 0	0.25 ± 0	–
The number of doses of GnRH antagonist, times, mean ± SD	1.0 ± 0	1.0 ± 0	1.000
Cost for ovulation suppression^c^, mean ± SD	USD9.1 ± 0 (JPY1,000 ± 0)	USD72.9 ± 0 (JPY8,000 ± 0)	**<0.001** ^a^
Total ovulation rate, cycle, n (%)	9 (40.9)	0 (0)	**0.002** ^b^
Clomiphene citrate + FSH/hMG	6 (54.5)	0 (0)	**0.012** ^b^
Letrozole + FSH/hMG	3 (27.3)	0 (0)	0.218^b^
Findings of retrieved oocytes			
No. of retrieved oocytes, n, mean ± SD	5.7 ± 2.8	5.6 ± 5.7	0.942^a^
No. of retrieved mature oocytes, n, mean ± SD	3.5 ± 2.6	4.0 ± 4.1	0.662^a^
Oocyte maturation rate /oocyte, %, mean ± SD	61.2 ± 36.1	81.4 ± 23.9	0.115^a^

Abbreviations: AMH, anti‐Müllerian hormone; SD, standard deviation.

Bold indicates statistical significance *p* < .05.

^a^ Student's *t*‐test.

^b^ Fisher's exact test.

^c^ Converted to USD from JPY using average USD exchange rate in September 2021 published by Bank for International Settlements (JPY109.8/USD).

### 3.3 Clinical outcomes of fertilization, embryo culture, and subsequent pregnancy

To confirm the effect of relugolix on retrieved oocytes, we also examined fertilization and subsequent embryo culture outcomes in 106 cycles in the relugolix group and 98 cycles in the injection group (Table 3). The fertilization rates after conventional IVF and ICSI did not differ between the two both groups (*p *= 0.511 and 0.560, respectively). In 145 cycles with performing blastocyst cultures, there were no significant differences in the formation rates of blastocysts and morphologically good blastocysts in the relugolix and injection groups (*p* = 0.882 and 0.401, respectively).

**TABLE 3:** Fertilization and embryo culture outcomes.

	Relugolix 106 cycles (106 women)	Injection 98 cycles (94 women)	*p*‐value
Conventional IVF	61 cycles (61 women)	39 cycles (36 women)	
No. of retrieved MII oocytes, n, mean ± SD	5.7 ± 3.7	6.7 ± 4.9	0.286^a^
Fertilization rate, %, mean ± SD	62.1 ± 32.2	66.4 ± 31.3	0.511^a^
ICSI, mean ± SD	45 cycles (45 women)	59 cycles (58 women)	
No. of retrieved MII oocytes, n, mean ± SD	5.6 ± 3.2	5.8 ± 4.2	0.801^a^
Fertilization rate, %, mean ± SD	76.9 ± 26.8	73.9 ± 25.2	0.560^a^
Blastocyst culture	78 cycles (77 women)	67 cycles (65 women)	
No. of embryos in blastocyst culture, n, mean ± SD	3.6 ± 2.1	4.1 ± 3.6	0.295^a^
No. of blastocysts, n, mean ± SD	2.3 ± 1.8	3.0 ± 3.0	0.129^a^
Blastocyst formation rate, %, mean ± SD	65.3 ± 37.5	66.3 ± 36.0	0.882^a^
No. of morphologically good blastocysts^b^, n, mean ± SD	1.7 ± 1.4	1.9 ± 2.2	0.626^a^
Morphologically good blastocyst formation rate, %, mean ± SD	48.7 ± 35.1	43.6 ± 37.3	0.401^a^

Abbreviations: ICSI, intracytoplasmic sperm injection; SD, standard deviation.

^a^ Student's *t*‐test.

^b^ Morphologically good blastocysts were defined as 5‐ or 6‐day blastocysts after fertilization except for grade C in either the inner cell mass or the trophectoderm of the Gardner classification.

The clinical pregnancy rates in the relugolix and injection groups were 25.5% (13/51 cycles) and 18.2% (8/44 cycles) after ET using cleavage embryos (*p* = 0.392) and 40.7% (33/81 cycles) and 45.8% (27/59 cycles) after ET with blastocysts (*p* = 0.596), respectively (Table 4). There were no significant differences in clinical pregnancy rates after ET cycles with either cleavage or blastocyst stage embryos (*p *= 0.392 and 0.596, respectively). The miscarriage rates also did not differ significantly between the two groups.

**TABLE 4:** Pregnancy outcomes after embryo transfer

	Relugolix 132 cycles (98 women)	Injection 103 cycles (78 women)	*p*‐value
Age, years, mean ± SD (range)	35.6 ± 3.6	36.7 ± 3.7	0.313^a^
ET cycles with cleavage stage embryos	51 cycles	44 cycles	
Fresh ET, n (%)	26 (51.0)	24 (54.5)	0.729^b^
Vitrified‐warmed ET, n (%)	25 (49.0)	20 (45.5)	
No. of transferred embryos, n, mean ± SD	1.2 ± 0.4	1.2 ± 0.5	0.963^a^
Clinical pregnancy rate, n (%)	13 (25.5)	8 (18.2)	0.392^b^
Miscarriage rate, n (%)	1 (7.7)	1 (12.5)	0.716^b^
ET cycles with blastocysts	81 cycles	59 cycles	
Fresh ET, n (%)	2 (2.5)	2 (3.4)	0.747^b^
Vitrified‐warmed ET, n (%)	79 (97.5)	57 (96.6)	
No. of transferred embryos, n, mean ± SD	1.0 ± 0.1	1.1 ± 0.3	0.076^a^
Clinical pregnancy rate, n (%)	33 (40.7)	27 (45.8)	0.596^b^
Miscarriage rate, n (%)	4 (12.1)	7 (25.9)	0.082^b^

Abbreviations: ET, embryo transfer; SD, standard deviation.

^a^ Student's *t*‐test.

^b^ Fisher's exact test.

## 4 DISCUSSION

This is the first study to examine the applicability of a novel oral GnRH antagonist agent, relugolix, as an ovulation inhibitor in IVF treatment. Oral relugolix (TAK‐385) can antagonize gonadotropin secretion from the pituitary in both women and men; therefore, it has been reported as a therapeutic medicine for uterine fibroids, endometriosis, and prostate cancer.^9^, ^10^, ^14^, ^15^, ^16^ Our study also showed relugolix has a high ovulation suppressive effect prior to the LH surge compared with injection preparations of GnRH antagonists. However, 40% or more of the cycles that used relugolix after the LH surge started had ovulation before oocyte retrieval.

From a pharmacokinetic perspective of GnRH antagonists, the time to maximum drug concentration in the serum (Tmax) of cetrorelix and ganirelix is approximately 1–1.5 h.^5^, ^7^ Conversely, the average Tmax of relugolix is 2.0 h.^17^ Relugolix also has a short Tmax, yet it has a slightly longer duration than those of cetrorelix and ganirelix. The Tmax of relugolix may be longer because of the required intestinal absorption after internal use compared with injection preparations. The average half‐lives (T1/2) of cetrorelix, ganirelix, and relugolix are 51.9, 16.2, and 36−65 h, respectively ^5^, ^7^, ^9^, ^17^; therefore, it is unlikely that the high ovulation rate was caused by a decrease in the effect of relugolix on ovulation suppression after relugolix use before oocyte retrieval. In pharmacodynamics, cetrorelix and ganirelix have displayed a high effectiveness for inhibiting LH levels in a short period (4−7 h) to the LH nadir.^5^, ^7^ After the intake of oral relugolix, the time to the LH nadir is approximately 12 h^17^; thus, the late onset of the inhibitory effect on LH levels may be responsible for the high ovulation ratio when using relugolix during the LH surge. Furthermore, the bioavailability of relugolix is reduced by diet^9^; therefore, relugolix should be consumed before meals. However, some patients had to take it immediately when the LH surge was confirmed; therefore, having meals prior to relugolix intake may decrease its suppressive effect of premature ovulation.

There were no significant differences in the findings of retrieved oocytes, fertilization, embryo culture, and subsequent pregnancy outcomes in the two groups that received either oral relugolix or injections of cetrorelix or ganirelix. Although we used mild ovarian stimulation protocols, relugolix can be used in a conventional GnRH antagonist protocol. Adverse events of long‐term administration of relugolix including headache and hot flush have been reported,^10^, ^14^ but there were no women who complained the symptoms of its side effects of short‐term relugolix treatment in our study. Oral relugolix is easier and safer to use and costs less compared with injection preparations; thus, before the LH surge occurs, it can be an alternative clinical application to GnRH antagonist injection, leading to reduced physical and financial burdens for patients. Recently, a novel ovarian stimulation protocol, progestin‐primed ovarian stimulation (PPOS) with gonadotropin injection in combination with oral progestin for ovulation suppression, has been reported.^18^, ^19^ PPOS has good advantages with high cost‐effectiveness^20^; however, all embryos must be frozen after this ovarian stimulation. Relugolix can be used in fresh ET cycles.

This study has some limitations. First, this is a single‐center, retrospective study. Second, only a small number of patients were included in this study of GnRH antagonist use during the LH surge because there were not many women with high LH levels on the day of oocyte maturation. Furthermore, we had to stop administering relugolix during the LH surge because of the high premature ovulation rate prior to oocyte retrieval. Third, clomiphene citrate has an LH surge suppressive effect, but the treatment cycles using clomiphene citrate were included in this study, because there was no significant difference in the rates of clomiphene use in relugolix and injection groups. In addition, of nine women who had premature ovulation after relugolix intake during LH surge, six women used clomiphene; thus, we included the cycles with clomiphene use. Fourth, we could not confirm the number of the cycles in the patients having meal before relugolix intake during LH surge.

In conclusion, relugolix had a high ovulation suppressive effect and no adverse effects on retrieved oocytes and subsequent pregnancy outcomes following ET in our study. Hence, the ovarian stimulation protocols using oral relugolix can be an alternative stimulation method with low costs and minimum invasion. However, when the LH surge was recognized in mild stimulation protocols, its effect was insufficient to prevent premature ovulation; thus, relugolix should be avoided in such cases. Additional clinical studies including prospective controlled trials in GnRH antagonist cycles are required to confirm the therapeutic effect of relugolix on premature ovulation and pregnancy outcomes in IVF treatment.

## CONFLICTS OF INTEREST

All authors have no conflicts of interest to declare relevant to this study. Human rights statement and informed consent: This study was approved by the local ethics committee of Sugiyama Clinic (No. 20–005). All procedures followed were in accordance with the ethical standards of the responsible committee on human experimentation and with the Helsinki Declaration of 1964 and its later amendments. All recruited women provided written informed consent.
